# A life-attenuated BLV deletant as a candidate vaccine to inhibit viral transmission in bovine herds

**DOI:** 10.1186/1742-4690-8-S1-A12

**Published:** 2011-06-06

**Authors:** Gerónimo Gutiérrez, Sabrina M Rodríguez, Arnaud Florins, Lucas Vagnoni, Irene Alvarez, Nicolas Gillet, Karina Trono, Luc Willems

**Affiliations:** 1 Instituto de Virología, CICVyA, Instituto Nacional de Tecnología Agropecuaria, Castelar, 1712, Argentina; 2 Molecular and Cellular Epigenetics, Interdisciplinary Cluster for Applied Genoproteomics (GIGA), University of Liège (ULg), Liège, 4100, Belgium; 3 Molecular and Cellular Biology, Gembloux Agro-Bio Tech, University of Liège (ULg), Gembloux, 5030, Belgium

## 

There are different strategies to reduce BLV prevalence. Eradication by culling of infected animals is not economically sustainable in highly infected regions such as Argentina, US or Japan. Segregation of BLV-infected cows is expensive due to duplication of facilities. Finally, several candidate vaccines based on recombinant viral proteins were unsuccessful to protect from challenge.

Facing these problems, we propose a novel strategy based on the use of a live-attenuated BLV provirus. The rationale behind this strategy relies on the deletion of genes required to induce pathogenesis leaving those involved in infectivity, resulting in an attenuated mutant with impaired transmissibility.

In a first set of experiments, we showed that the mutant is infectious and elicits an efficient immune response in sheep (n=3) and in cows (n=9). Lack of spread to uninfected sentinels further supports the safety of the vaccine. Based on these promising results, a validation program in herd (n=105) is ongoing to evaluate the capacity of the candidate vaccine to protect from wild-type BLV infection. The following experiments are carried out: quantification of the proviral loads, assessment of immune response efficiency (antibody titers, CTL response and cytokine profiling), measure of viral expression in vivo (qRT-PCR) and ex vivo (expression of Tax and p24gag) and determination of provirus clonality during infection.

This data will be instrumental for understanding the basic mechanisms undergoing during BLV infection and for elaborating a novel vaccine.

